# Review of microRNA detection workflows from liquid biopsy for disease diagnostics

**DOI:** 10.1017/erm.2025.2

**Published:** 2025-02-06

**Authors:** Dulguunnaran Naranbat, Emilia Herdes, Nikos Tapinos, Anubhav Tripathi

**Affiliations:** 1 Center for Biomedical Engineering, School of Engineering, Brown University, Providence, RI, USA; 2 Warren Alpert Medical School, Brown University, Providence, RI, USA; 3 Department of Neurosurgery, Rhode Island Hospital, Providence, RI, USA

**Keywords:** disease diagnostics, liquid biopsy, microRNA, miRNA

## Abstract

MicroRNAs have emerged as effective biomarkers in disease diagnostics, particularly cancer, due to their role as regulatory sequences. More recently, microRNAs have been detected in liquid biopsies, which hold immense potential for early disease diagnostics. This review comprehensively analyses distinct liquid biopsy microRNA detection methods validated with clinical samples. Each step in the microRNA detection workflow, including sample collection, RNA isolation, processing, and detection of target microRNAs, has been thoroughly assessed. The review discusses the advantages and limitations of established and novel techniques in microRNA detection workflows, discussing their diagnostic capabilities and potential for future implementation at scale.

## Introduction

In recent decades, a new frontier in disease diagnostics, particularly for cancer, has emerged with microRNA (miRNA) detection. Numerous researchers have underscored the pivotal role of specific miRNAs in the onset of tumorigenesis, suggesting their potential as diagnostic biomarkers. (Refs 1, 2, 3) miRNAs are short, non-coding RNA sequences, 19–25 nucleotides long, that play a crucial role in post-transcriptional gene regulation. (Refs 4, 5) The maturation of functional miRNAs begins with a long single-stranded RNA that forms a hairpin structure called primary miRNA (pri-miRNA). In the nucleus, the pri-miRNA is cleaved by an enzyme complex into precursor miRNA (pre-miRNA), which is then exported to the cytoplasm. An enzyme cleaves it into a double-stranded RNA, which unwinds to produce mature miRNA. Mature miRNAs bind to target messenger RNAs, modulating gene expression and contributing to disease progression. (Refs 4, 6) Numerous target miRNAs have been identified to exhibit either overexpression or underexpression in association with specific diseases. (Ref. 4) Therefore, once the correlation between specific miRNAs and disease is established, the abundance of the target miRNA in affected individuals can be measured and compared to controls, serving as a diagnostic marker. (Refs 4, 7)

Currently, cancer diagnosis predominantly relies on tissue biopsies. While effective, these biopsies are invasive, often difficult to access (such as in brain tumours), and not well-suited for continuous monitoring. (Refs 8, 9, 10, 11) As a result, miRNA detection through liquid biopsies - such as blood, urine, and saliva - has become a rapidly advancing area of research. This progress enhances the development of cost-effective and time-efficient point-of-care (POC) testing systems for miRNA detection, with significant potential to improve early disease screening and diagnosis. (Ref. 12)

**Figure 1.**
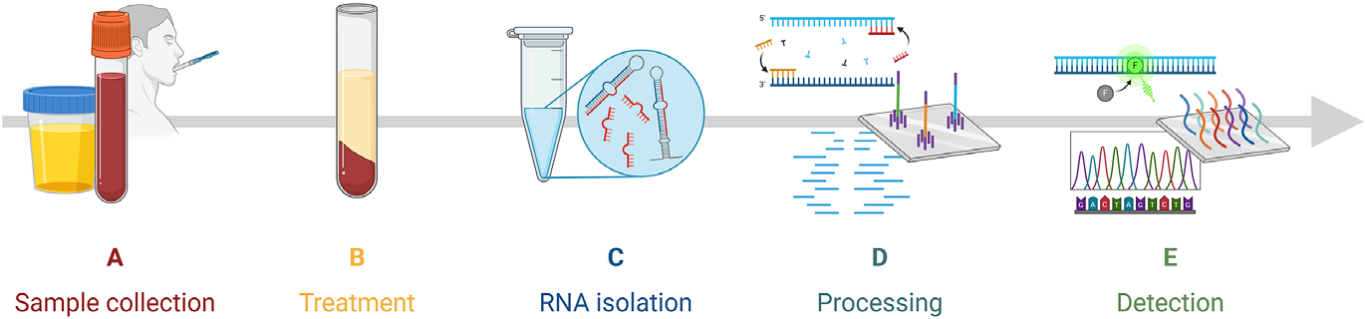
General overview of miRNA detection workflow. The steps are as follows: A. Sample collection from the patient, B. sample treatment, C. small RNA isolation including miRNA, D. sample processing including methods of target amplification, library preparation, and surface hybridisation, and E. detection of signature miRNA.

**Figure 2.**
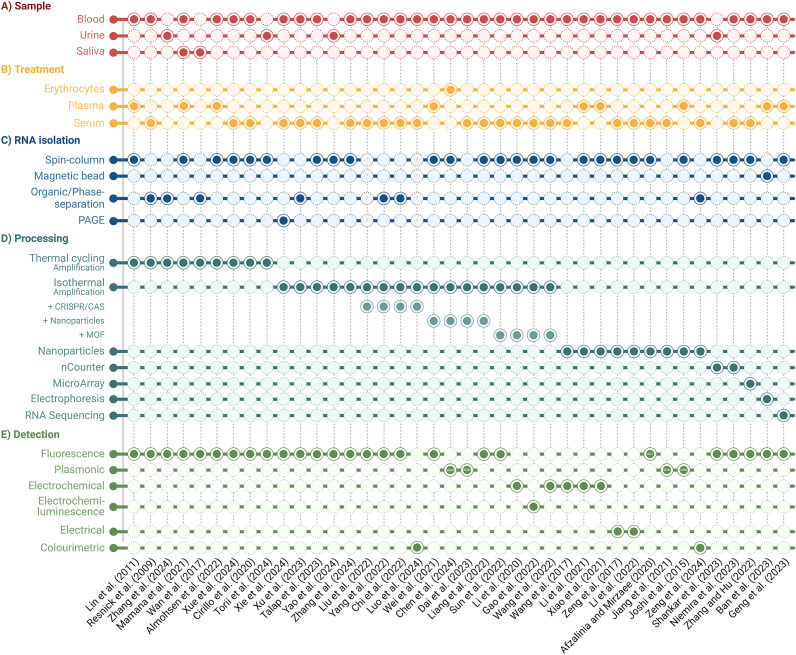
Comparison of clinically validated miRNA detection workflows. Sample type, treatment, RNA isolation, processing, and detection methods are outlined.

While miRNAs in liquid biopsy show promise as disease biomarkers, their practical applicability is still under evaluation. Successful implementation necessitates a standardised workflow from sample collection to detection [Figure 1 **]**. The absence of standardised protocols and guidelines for extracting, quantifying, and normalising miRNA levels has significantly hindered the reliability and validity of miRNA detection methods as they transition from basic research to clinical applications. (Refs 13, 14) Novel detection methods are continually advancing, but validation with clinical data is crucial to establish their feasibility. This review comprehensively analyses distinct miRNA detection methods from liquid biopsies validated with clinical samples. Here, we examine each step in the miRNA detection workflow, including sample collection, RNA isolation, processing and detection of target miRNAs [Figure 2]. A comprehensive list detailing each study’s workflow and the reported limit of detection (LOD) is provided in Table 1. The figures in this review were made with BioRender.com (Toronto, Canada).

**Table 1.** Overview of unique miRNA detection workflow for targeted disease diagnostics from liquid biopsies

Processing method	Detection method	Input sample	RNA isolation method	RNA isolation kit	Processing and detection kit	miRNA target	Disease target	Reported LoD	Authors/*Vendor*
**Thermal cycling amplification** - Stem-loop RT-qPCR	**Fluorescence** – SYBR Green	Plasma	Spin column	miRNeasy Serum/Plasma Kit (Qiagen)	SYBR Green qPCR SuperMix-UDG (Invitrogen)	miR–125a–5p, miR–126, miR–183, miR–200, miR–221, and miR–222	Lung Cancer	NDNR (SOP – 10 miRNA molecules)	Lin et al. (2012)
**Thermal cycling amplification** - Stem-loop RT-qPCR	**Fluorescence** – Probe based	Serum	Organic phase separation	Tri-Reagent BD (Molecular Research Center, Inc.)	TaqMan MicroRNA Assays (Applied Biosystems)	miR–21, miR–16, miR–29a, let–7c, let–7f, miR–125a, miR–125b, miR–126, miR–133, and miR–93	Ovarian cancer	NDNR (SOP – 10 miRNA molecules)	Resnick et al. (2009)
**Thermal cycling amplification** - Stem-loop RT-qPCR	**Fluorescence** – Probe based	Urine	Organic phase separation	TRIzol reagent (Invitrogen)	TaqMan MicroRNA Assays (Applied Biosystems)	miR–135b–5p, miR–196b–5p, miR–200c–3p, and miR–203a–3p	Early-stage renal cell carcinoma	NDNR (SOP – 10 miRNA molecules)	Zhang et al. (2024)
**Thermal cycling amplification** - Stem-loop RT-qPCR	**Fluorescence** – Probe based	Saliva & Plasma	Spin column	Saliva/Swab RNA Purification Kits (Norgen)	TaqMan MicroRNA Assays (Applied Biosystems)	miR–5p	Polyomavirus	10 copies of viral miRNA–5p per reaction	Mamana et al. (2021)
**Thermal cycling amplification** – Poly(A)-tailing/ Stem-loop RT-qPCR	**Fluorescence** – Probe based (Universal or miRNA specific)	Bacteria, Virus, Cells, Plant, Tissue, Yeast	Organic phase separation	PureLink/ mirVana miRNA Isolation Kit (Invitrogen)	TaqMan (Advanced) MicroRNA Assays (Applied Biosystems)	Custom	Custom	Target dependent	*ThermoFisher Scientific*
**Thermal cycling amplification** - Stem-loop RT-qPCR	**Fluorescence** – SYBR Green/ Probe based	Tissue, Cells	Spin column	miRNeasy Mini Kit (Qiagen)	miRCURY LNA RT kit + miRCURY LNA SYBR Green PCR Kits/ miRCURY LNA miRNA PCR Assays (Qiagen)	Custom	Custom	Target dependent	*Qiagen*
**Thermal cycling amplification** - Poly(A) tail RT-qPCR	**Fluorescence** – SYBR Green	Saliva	Organic phase separation	NucleoSpin miRNA kit (Machnery-Nagel)	miRNA miScript primer PCR Assays (Qiagen)	9 miRNAs	HPV-positive head and neck squamous cell carcinomas	NDNR (60% compared to healthy controls)	Wan et al. (2017)
**Thermal cycling amplification** - Poly(A) tail RT-qPCR	**Fluorescence** – SYBR Green	Platelet-poor plasma	Spin column	miRNeasy Serum/Plasma Kit (Qiagen)	miRCURY LNA SYBR Green PCR Kit (Qiagen)	miR–126–3p and miR–423–5p	Acute myeloid leukaemia	NDNR (Ct values compared; SOP – 10 miRNA molecules)	Almohsen et al. (2022)
**Thermal cycling amplification** - SMOS RT-qPCR	**Fluorescence** – SYBR Green	Serum	Spin column	Versamedx(r) miRNA extraction kit (Suzhou VersaBio Technologies Co.)	*Custom*	hsa-miR–16, miR–93, miR–21, and cel-miR–39	Esophageal cancer	0.1 zM	Xue et al. (2024)
**Thermal cycling amplification** - ddPCR	**Fluorescence** – SYBR Green	Serum	Spin column	miRNeasy SerumPlasma Advanced Kit (Qiagen)	EvaGreen Supermix (BioRad)	miR–149–3p and miR–320a	Ovarian cancer	cel-miR–39–3p was 1.12–0.16 copies/uL	Cirillo et al. (2020)
**Thermal cycling amplification** - ddPCR	**Fluorescence** – Probe	Urine	Spin column	EVery EV RNA Isolation Kit (System Biosciences)	miRCURY LNA miRNA PCR Assay (Qiagen)	6 miRNAs	Congenital cytomegalovirus (cCMV) infection	NDNR (SOP – 10 miRNA molecules)	Torii et al. (2024)
**Isothermal amplification** - RCA + APE–1	**Fluorescence** – Dual	Serum	Gel electrophoresis	Bio-Rad electrophoresis system	*Custom*	miR–206	Alzheimer’s disease	1.82 fM	Xie et al. (2024)
**Isothermal amplification** - Padlock Assisted HRCA	**Fluorescence** – SYBR Green	Serum	Organic phase separation	miRNA First Strand cDNA Synthesis Kit (Agilent)	*Custom*	miR–10b and miR–155	Liver cancer and breast cancer.	133.9 aM	Xu et al. (2023)
**Isothermal amplification -** PS-LAMP	**Fluorescence** – SYBR Green	Serum	Spin column	miRNeasy Serum/Plasma Advanced Kit (Qiagen)	*Custom*	miR–146b–5p and miR–199b–5p	Papillary thyroid cancer	1.0 aM	Talap et al. (2023)
**Isothermal amplification -** CDiPER	**Fluorescence** – Probe based	Serum	Spin column	miRNeasy RNA Isolation Kit (Qiagen)	*Custom*	miR–21	Diabetes mellitus	312 aM	Zhang et al. (2024)
**Isothermal amplification -** MEXPAR	**Fluorescence**	Urine	Spin column	miRcute miRNA Isolation Kit (Applied Biosystems)	*Custom* - Smartphone-based imaging	let–7a, miR–155, miR–223, and miR–143	Prostate, bladder, and renal cell cancers	59–1300fM (=0.059–1.3pM)	Yao et al. (2024)
**Isothermal amplification + CRISPR/Cas -** RCA + CRISPR/Cas9	**Fluorescence**	Serum	N/A	N/A	*Custom*	miR–326	Lung cancer with brain metastasis	3.45 fM	Liu et al. (2022)
**Isothermal amplification + CRISPR/Cas -** EXPAR + CRISPR/Cas12a	**Fluorescence** – Probe based	Serum	Organic phase separation	Centrifugation	*Custom*	miR–27a	Breast cancer	90 aM	Yang et al. (2022)
**Isothermal amplification + CRISPR/Cas -** SDA + CRISPR/Cas14a	**Fluorescence** – Probe based	Serum	Organic phase separation	Trizol/ Centrifugation	*Custom*	miR–21	Cholangiocarcinoma	680 fM	Chi et al. (2022)
**Isothermal amplification + CRISPR/Cas -** ttSDA + CRISPR/Cas12a + Pt@Au nanozymes	**Colourimetric**	Serum	N/A	N/A	*Custom*	miR–21	Hepatocellular carcinoma	1 pM – 500 pM	Luo et al. (2024)
**Isothermal amplification + Nanoparticles** - HCR + MOS2 nanosheets	**Fluorescence**	Plasma	Spin column	*Custom*	*Custom*	miR–21	Lung cancer	6 pmol/L	Wei et al. (2021)
**Isothermal amplification + Nanoparticles** - HCR + Fe3O4 magnetic nanospheres	**Fluorescence**	Serum	Spin column	Total RNA rapid extraction kit (Shanghai Shenggong Biotechnology)	*Custom*	miR–182	Glioma	20 fM	Liang et al. (2022)
**Isothermal amplification + Nanoparticles** -CHA + HCR + Au@Ag nanoparticles	**Plasmonic** – SERS	Erythrocytes	Spin column	SanPrep Column microRNA Extraction Kit (Sangon)	*Custom*	miR–210	β-thalassemia	1.17 pM	Chen et al. (2024)
**Isothermal amplification + Nanoparticles -** CHA + EASA + Au-Ag nanoshuttles	**Plasmonic** – SERS	Serum	N/A	N/A	*Custom*	miR–122 and miR–192	Colorectal cancer	4.26 aM	Dai et al. (2023)
**Isothermal amplification + MOF** - RCA + MOF–525	**Fluorescence**	Serum	Spin column	miRNeasy Micro Kit (Qiagen)	*Custom*	miR–92a–3p	Colorectal cancer	0.03 pM	Sun et al. (2022)
**Isothermal amplification + MOF** - PER+ MOF@Pt@MOF nanonzyme	**Electrochemical**	Serum	Spin column	Universal miRNA extraction kit (EZBioscience)	*Custom*	miR–21	Breast cancer	0.29 fM	Li et al. (2020)
**Isothermal amplification + MOF** - RHOA + Fe-Zr MOF/G4 nanozyme	**Electrochemiluminescence**	Serum	Spin column	Exosome Isolation Kit (KeyGEN BioTECH)	*Custom*	miR–15a–5p	Endometrial cancer	1.59 aM	Gao et al. (2022)
**Isothermal amplification + MOF** - λ-exonuclease + ZIF–67 MOF/WO3 nanoflakes	**Electrochemical**	Serum	Spin column	exoRNeasy Serum/Plasma Midi Kit (Qiagen)	*Custom*	miR–21	Breast cancer	0.5 fM	Wang et al. (2022)
**Nanoparticles -** Magnetic microbeads & diblock oligonucleotide-modified Au nanoparticles	**Electrochemical**	Serum	N/A	N/A	*Custom*	miR–182 and miR–381	Glioma (brain tumour)	0.20 fM and 0.12 fM	Wang et al. (2017)
**Nanoparticles -** Peptide nucleic acid functionalized nanochannel biosensor	**Electrochemical**	Plasma	Spin column	exoRNeasy Serum/Plasma Midi Kit (Qiagen)	*Custom*	miR–10b	Pancreatic cancer	75aM	Xiao et al. (2021)
**Nanoparticles -** DNA tetrahedral nanostructure-based biosensor	**Electrochemical**	Serum	Spin column	miRNeasy RNA isolation kits (Qiagen)	*Custom*	miR–21, miR–55, miR–196a, and miR–210	Pancreatic Carcinoma	10 fM	Zeng et al. (2017)
**Nanoparticles -** Carbon nanotube field-effect transistor biosensor	**Electrical**	Plasma	Spin column	exoRNeasy Serum/Plasma Midi Kit (Qiagen)	*Custom*	miR–21	Breast cancer	0.87 aM	Li et al. (2021)
**Nanoparticles -** PMO-graphene quantum dots biosensor	**Electrical**	Serum	Spin column	miRNeasy RNA isolation kits (Qiagen)	*Custom*	miR–21	Breast cancer	85 aM	Li et al. (2022)
**Nanoparticles -** La(III)-MOF & Ag nanoparticle biosensor	**Fluorescence** – FRET	Serum	Spin column	miRNeasy RNA isolation kits (Qiagen)	*Custom*	miR–155	Lung & breast cancer	5.5fM/ 0.04 ppb	Afzalinia and Mirzaee (2020)
**Nanoparticles -** Fe3O4@TiO2 nanoparticle biosensor	**Plasmonic** – SERS	Plasma	Spin column	TRIzol kit w/ Direct-zol RNA MiniPrep kit (Zymo Research)	*Custom*	miR–10b	Pancreatic cancer	32.6 aM	Jiang et al. (2021)
**Nanoparticles -** Au nanoprism biosensor	**Plasmonic** – LSPR	Serum	N/A	N/A	*Custom*	miR–10b	Pancreatic ductal adenocarcinoma	0.21 fM	Joshi et al. (2015)
**Nanoparticles -** Au nanoparticle liposome-based microfluidic chip biosensor	**Colourimetric**	Serum	Organic phase separation	*Custom*	*Custom* - Smartphone camera	miR–21	Diabetes (Type II)	0.27 pM	Zeng et al. (2024)
**nCounter -** Nanostring	**Fluorescence**	Urine	Spin column	Urine Exosome RNA isolation kit (Norgen)	nCounter Analysis System (NanoString Technologies)	799 miRNAs	IgA nephropathy	NDNR (Detection compared; SOP 0.5 fM)	Shankar et al. (2023)
**nCounter –** Nanostring	**Fluorescence**	Serum	Spin column	miRNeasy SerumPlasma Advanced Kit (Qiagen)	nCounter (NanoString)	miR–1246 and miR–150–5p	Epithelial ovarian cancer	NDNR (Detection compared; SOP 0.5 fM)	Niemira et al. (2023)
**RNA Sequencing -** Next generation sequencing (NGS)	**Fluorescence**	Plasma	Spin column	miRNeasy SerumPlasma kit (Qiagen)	MiSeqDx (Illumina)	17 miRNAs	Non-small cell lung cancer	NDNR (SOP 25 ng of RNA input)	Geng et al. (2023)
**RNA Sequencing -** Next generation sequencing (NGS)	**Fluorescence**	Serum, Plasma, FFPE	Organic/ Probe based	Custom	EdgeSeq (HTG)	Custom	Custom	Target dependent	*Abcam*
**Flow cytometry**	**Fluorescence**	Serum, Plasma, Urine, Saliva, FFPEs	Custom	N/A	FirePlex miRNA Assay (abcam)	Custom	Custom	Target dependent	*HTG*
**Microarray**	**Fluorescence**	Serum	Spin column	RNeasy 96 QIAcube HT Kit (Qiagen)	3D-Gene Human miRNA Oligo Chip	50 miRNAs	12 cancers	NDNR (Detection compared; SOP 0.1 aM)	Zhang and Hu (2022)
**Capillary electrophoresis**	**Fluorescence**	Plasma	Magnetic beads	*Custom*	*Custom*	miR–21	Lung cancer	58 fM	Ban et al. (2023)

*Note*: Normalised data not reported is abbreviated as NDNR.

## Liquid biopsy samples and treatment

Numerous studies suggest a correlation between miRNA levels in whole blood and tissue samples, presenting an opportunity to develop a less invasive method for accurate target miRNA detection. (Refs 15, 16, 17) This review focused on miRNA detection methods using blood, saliva, and urine samples.

### Peripheral blood

Blood is the most commonly used liquid biopsy sample for detecting miRNA due to its higher concentration of detectable miRNAs than other liquid biopsies. (Refs 7, 18, 19, 20) Evidence suggests that miRNAs circulate in peripheral blood within cells, encapsulated in extracellular vesicles (EVs), or as cell-free molecules. Furthermore, miRNAs encapsulated in protein complexes or EVs gain stability, making them ideal biomarkers. (Ref. 21) Therefore, blood-based miRNA detection represents a promising frontier but necessitates standardised extraction methods to prevent procedural variations from affecting miRNA levels. (Ref. 22) EV-derived miRNAs have demonstrated better diagnostic capabilities in some cases; for example, target miRNAs – miR-200c-3p and miR-21-5p have performed better in distinguishing patients with prostate cancer when analysed in plasma EVs compared to whole plasma with the potential to be a consistent source of RNA. (Ref. 23) However, this may not be true depending on the specific target miRNA or pathological condition, as seen with miR-375, which showed better detection performance in whole plasma compared to EVs. (Ref. 23)

**Figure 3.**
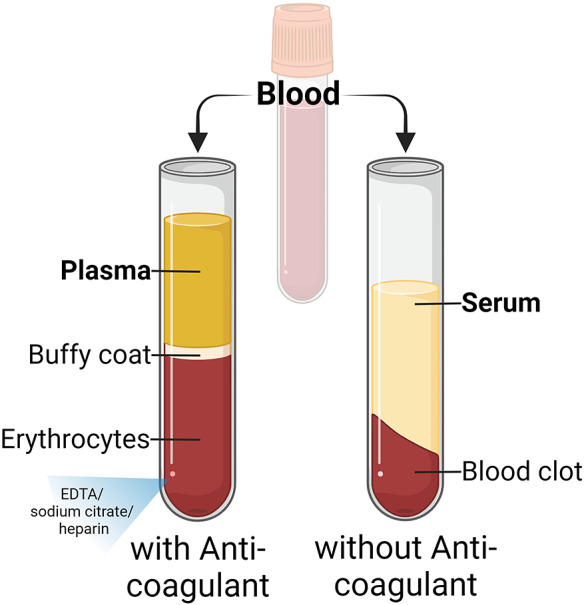
Blood treatment for plasma and serum separation. Plasma is obtained by centrifugation and treatment with an anticoagulant, such as EDTA, sodium citrate, or heparin. The serum is obtained by extracting the remaining liquid after clotted blood.

Peripheral blood is typically collected in vacutainer tubes and processed differently based on the application. Plasma or serum is ideal for detecting miRNA as it contains miRNA encapsulating EVs and has fewer contaminants than whole blood [Figure 3] (Refs 24, 25, 26, 27). Plasma is obtained by adding an anticoagulant and centrifugation. In contrast, serum is obtained by clotting the blood and extracting the liquid layer. Both contain EVs and platelets, which influence miRNA concentration. Filtering out platelets after centrifugation improves the quality and reproducibility of EV-miRNA analysis. (Ref. 28)

Anticoagulants like EDTA and sodium citrate are essential for preserving blood components before plasma separation. (Ref. 29) However, studies on miRNA detection in patients with cardiovascular diseases have found that administering heparin can affect miRNA concentrations in the blood, complicating the normalisation of miRNA levels. (Refs 30, 31, 32) Furthermore, heparin can co-isolate with RNA, interfering with downstream applications like qPCR, and thus is not recommended in these cases. (Ref. 33) Interestingly, there have been reports of the miRNA levels in serum remaining more stable over time compared to plasma, suggesting that samples without anticoagulants may be more suitable for miRNA detection. (Refs 34, 35) One significant concern with plasma and serum samples is hemolysis, a process where blood cells release their contents, including miRNAs, potentially affecting measured miRNA levels. (Refs 36, 37) The impact of hemolysis varies among different miRNAs, with some exhibiting greater stability in blood than others. For instance, miR-371-3p, a biomarker for testicular germ cell tumour detection, has been shown to be affected by hemolysis. (Refs 36, 37)

While target miRNA concentration levels may differ depending on overall pathological conditions, the concentration of miRNA in serum samples has been observed to be higher than in plasma samples. (Ref. 38) This difference may be linked to the coagulation process affecting extracellular miRNA in blood samples. (Ref. 38) Additionally, a case study of miRNA expression from patients with acute myocardial infarction showed that circulating miRNA concentrations in plasma and serum samples differ, indicating that research findings using distinct biological materials cannot be directly compared. (Ref. 31) Common reference genes such as RNU44, RNU48, and MammU6 were used to normalise target miRNA expression levels, but they also showed variation across different sample types, leading to inconsistency. (Ref. 38)

Plasma and serum have been the most common sample types for miRNA detection after blood extraction. However, Chen (Ref. 117) demonstrated that erythrocytes could also be used to detect miRNA associated with β-thalassemia, a blood disorder that affects haemoglobin. (Ref. 39) Despite lacking a nucleus and enclosed miRNAs, erythrocytes can carry cell-free miRNAs originating from various tissues circulating in the blood. (Ref. 40) While erythrocytes are likely not a suitable sample for all target miRNAs, they are used effectively in conditions that directly impact erythrocytes.

### Urine, Saliva and other Bodily Fluids

Although blood is the most extensively tested liquid biopsy sample for miRNA detection, successful detection from saliva and urine samples in clinical samples has also been achieved. While blood collection is less invasive than tissue biopsy, saliva and urine collection is non-invasive. However, the range of detected miRNAs in saliva and urine is generally less than half of those in the blood, limiting their use to specific diseases. (Ref. 20)

Free from protein contamination, urine samples have been effectively used to detect kidney, prostate, and bladder diseases through miRNA detection. (Refs 41, 42, 43, 44) In this review, cases of detection methods, including thermal cycling amplification, isothermal amplification and nCounter, have been discussed in detecting miRNA from urine.

While El-Mogy (Ref. 20) found that saliva contains the lowest range of detectable miRNAs, saliva samples have been successfully used to detect polyomavirus, human papillomavirus, and head and neck squamous cell carcinomas in clinical samples, as these viruses and cancers affect the oral cavity, allowing discernible differences in salivary miRNAs to be detected. (Ref. 20), (Refs 45, 46) Despite the potential use, limited clinical studies have used salivary miRNAs with novel processing methods due to the low range of miRNAs in saliva.

Other bodily fluids, such as cerebrospinal fluid (CSF) and pleural fluid, have also been effectively utilised as sources of miRNAs in patient samples. (Refs 47, 48) Unlike blood collection, obtaining CSF necessitates a lumbar puncture, while acquiring pleural fluid requires thoracentesis. (Refs 49, 50) Both procedures are significantly more invasive, demand specialised training, and carry higher risks and potential side effects compared to standard blood draws. While studies using other bodily fluids as liquid biopsies for clinical research are currently limited, they represent an emerging research direction in cancer detection.

It is important to note that standardisation and pre-analytical normalisation are crucial when comparing the capacity of detection methods from different liquid biopsy sample types. Multiple different procedures for establishing a consistent workflow have been suggested by ensuring standardised operating procedures (SOPs) for miRNA sample collection and detection. These procedures often improve reproducibility and provide quality control measures to help minimise variables during sample collection and testing. Variables such as collection method, turn-around time, anti-coagulation (such as avoiding heparin), storage conditions, volume, or formalin fixation options should all be considered. (Refs 51, 52) Given the sensitive nature of RNA, internal standardisation, such as freeze–thaw cycles, sample cold-chain systems, or qualitative assessments, such as hemolysis rate, are important factors when validating results. Depending on the sample type, different endogenous controls or spike-ins should be used for normalisation as reference genes here. (Ref. 53)

## RNA isolation methods

After sample collection and treatment, all small RNA, including miRNAs, are usually isolated through extraction and purification procedures [Figure 1]. Several different isolation methods exist as commercially available kits for this process. The studies examined in this review utilise various miRNA extraction techniques, including commercial kits or modified protocols.

### Organic phase separation-based extraction

**Figure 4.**
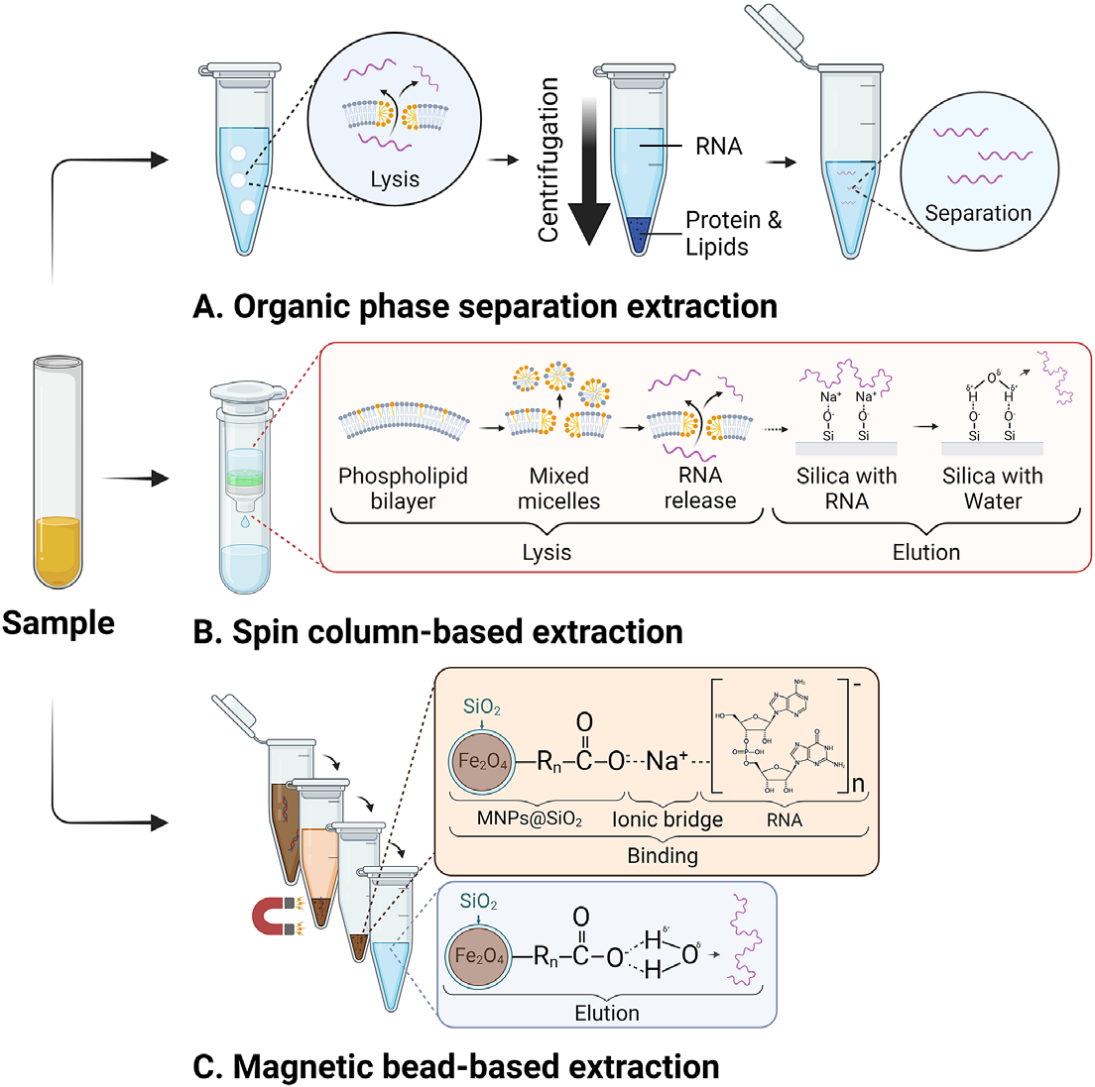
Overview of the three common types of RNA isolation for miRNA detection. A. Organic phase separation-based extraction method where vesicles are lysed, and contaminants are separated by centrifugal force. B. Spin column-based extraction method with a similar lysis procedure but where RNA is bound and eluted through a silica filter and C. Magnetic bead-based extraction method where RNA is bound to silica-based magnetic nanoparticles through ionic bridges supported by cations. The RNA is eluted sequentially in a water-based solution. (*Washing steps not shown*).

Organic phase separation-based extraction is a widely used RNA isolation method. (Ref. 54) Here, the method often employs phenol-containing solutions to lyse cells and vesicles, separating RNA from other cellular components. During lysis, often freely existing amphiphilic detergents create spherical micelles that insert themselves between the lipid molecules of the vesicle. This weakens the interactions that hold the bilayer together, thereby releasing nucleic acids from the vesicle [Figure 4A] (Ref. 55). Subsequently, the lysed samples are centrifuged to produce three distinct layers: 1) a lower organic phase, 2) an interphase containing unwanted protein and remaining DNA, and 3) an upper aqueous phase containing the RNA of interest. (Ref. 56) After isolating the aqueous phase, numerous centrifugation and washing steps are required to purify the small RNA. Adding glycogen and tRNA to the aqueous phase of plasma improves the extraction efficiency due to their roles as carrier molecules, which aid in forming precipitates that trap miRNA. (Refs 57, 58) While effective, the organic-phase separation method is manually intensive, laborious, and contains caustic and corrosive materials that must be handled cautiously. (Ref. 59) These factors increase the risk of errors and contamination, especially with larger sample sizes that demand more manual processing. (Ref. 59)

### Spin column-based extraction

The spin column-based extraction method provides a safer and simpler alternative to organic extraction for miRNA isolation. (Ref. 57) Similar to organic extraction, a lysis buffer is also used to lyse membranes. However, instead of organic solvents, it employs a silica membrane column and centrifugal force to capture RNA while allowing impurities to flow through [Figure 4B]. Due to the centrifugation requirement, the method remains challenging to automate, costly, and time-consuming. (Ref. 60) The miRNeasy Serum/Plasma Kit (Qiagen) has consistently demonstrated superior miRNA recovery in terms of quality and quantity compared to other commercial spin column-based kits available. (Refs 61, 62).

### Magnetic bead-based extraction

Magnetic bead-based small RNA isolation employs a unique solid-phase reversible immobilisation (SPRI) bead technique. (Ref. 63) Silica-based carboxyl-coated magnetic beads capture small RNA molecules from lysates, aided by a binding buffer containing polyethylene glycol (PEG) or alcohol, salt, and RNase inhibitors. PEG and alcohol create a “crowding” effect, forcing nucleic acids to cluster and intensify their negative charge. (Ref. 64) Additionally, the salts’ cations attract the negatively charged phosphates of the nucleic acid, creating an ionic bridge between carboxylated SPRI beads. (Refs 65, 66) Selection of small RNAs from the sample is often achieved by modifying the ratio of PEG and isopropanol during SPRI clean-up [Figure 4C] (Ref. 67). Magnetic bead-based extraction is advantageous due to its relatively low cost and quicker processing time, yet is limited due to its comparatively lower nucleic acid recovery rates. (Ref. 60) However, Timmerman (2021) reported that at low-volume inputs, the magnetic bead-based method outperformed the spin-column based miRNeasy Serum/Plasma Kit (Qiagen) (Ref. 62).

### Other extraction methods

Preparative polyacrylamide gel electrophoresis (PAGE) is another RNA isolation method in which input samples are loaded onto a polyacrylamide gel, and an electric current separates the RNA by size. (Ref. 68) In PAGE, a denaturing condition in the gel disrupts the hydrogen bonds formed by the nucleotides of the RNA input, allowing the RNA to migrate evenly by the size-sieving effect of the gel matrix. PAGE has been successfully utilised by Xie (Ref. 69) to detect miRNA from serum. (Ref. 69)

Interestingly, some recent studies have explored methods that successfully bypass RNA isolation altogether. (Refs 70, 71, 72) These methods are discussed further in the *Processing and Detection methods* section. If miRNA detection workflows without extraction achieve accuracy and sensitivity comparable to those with extraction, this advancement could significantly advance the development of POC testing platforms by drastically simplifying the entire detection process.

Extraction of miRNA exclusively from EVs is sometimes favoured because the target miRNAs may be more specific to EVs. (Ref. 73) Hence, several procedures, either prior to extraction or with combination extraction methods, have been utilised to extract EV-miRNA. Common techniques include ultracentrifugation, which is considered the gold standard for EV isolation, size-exclusion chromatography, and immunoaffinity capture processes, all of which have been reported to extract EV-miRNA efficiently. (Refs 74, 75, 76, 77) Multiple different commercial exosome isolation kits and exosome RNA isolation kits have also been made available for miRNA downstream processes, such as Total Exosome RNA & Protein Isolation Kit by Invitrogen (Waltham, MA) or RNeasy Plus Kits for RNA Isolation by Qiagen (Germantown, MD).

## Processing and Detection methods

### Thermal cycling amplification

The most common method of thermal cycling amplification is reverse transcription-quantitative polymerase chain reaction (RT-qPCR). RT-qPCR is considered the gold standard for detecting small RNAs, including miRNAs, due to its cost-effectiveness, sensitivity, specificity, and large dynamic input range. (Ref. 78) Two versions of miRNA specific RT-qPCR methods are discussed here.

#### Stem-loop RT-qPCR

**Figure 5.**
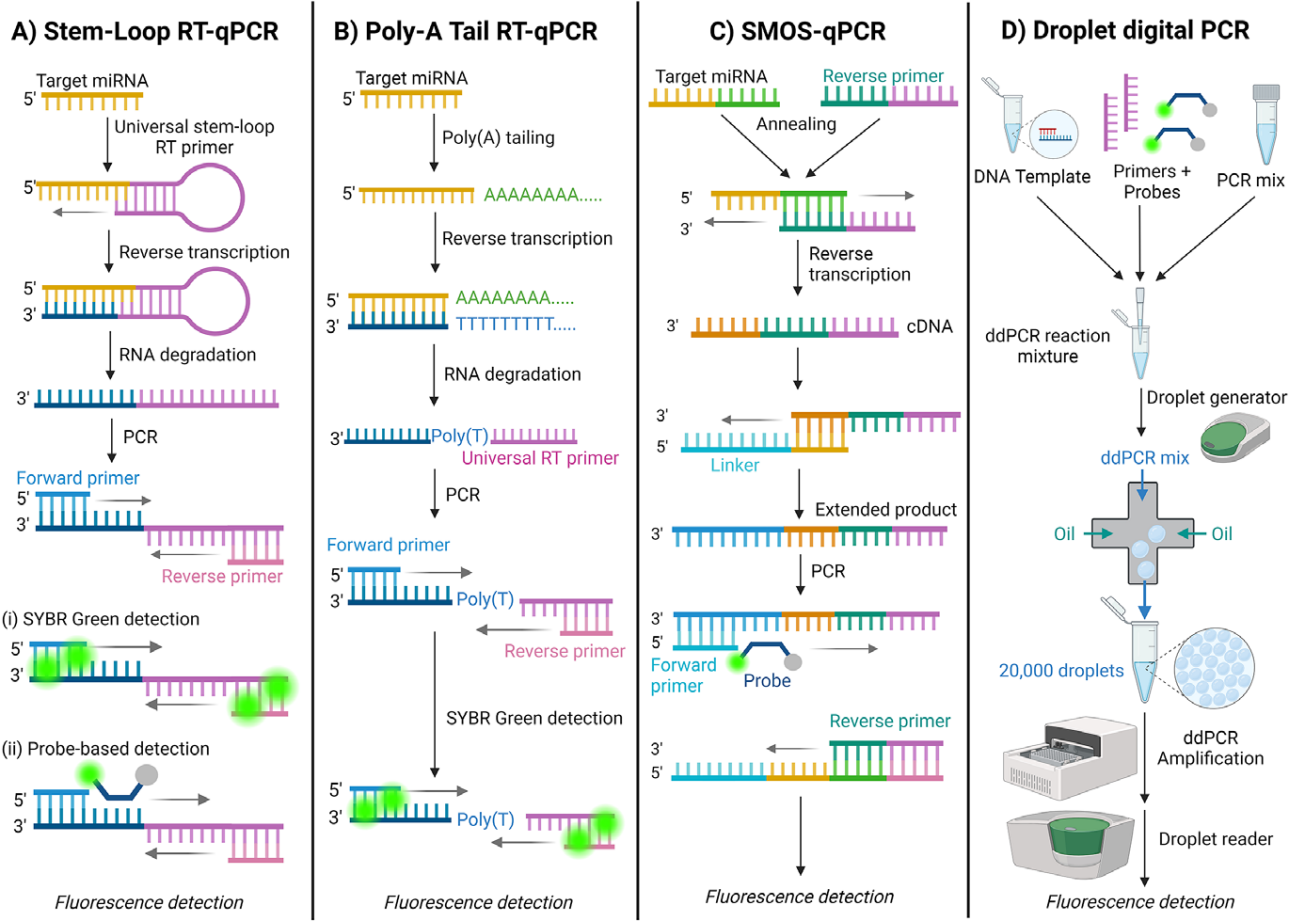
Schematic illustration of thermal amplification methods for miRNA detection. A. Stem-Loop RT-qPCR (Refs 41, 46, 79, 83, 84) with (i) SYBR Green detection - universal and (ii) Probe-based detection - specific; B. Poly-A Tail RT-qPCR; (Refs 45, 86) C. Sensitive and Multiplexed One-Step RT-qPCR (SMOS-qPCR); (Ref. 82) and D. Droplet digital PCR (ddPCR). (Refs 88,92)

Stem-loop RT-qPCR employs a universal stem-loop RT primer binding at the 3′ end of the miRNA template. (Ref. 79) The RNA secondary structure is melted and elongated in the RT thermal cycler to generate complementary DNA (cDNA) strands. These are then amplified using miRNA-specific forward and reverse primers [Figure 5A]. PCR inhibition by reverse transcriptase is common and is due to low template concentration; thus, cDNA strands must be diluted before being added to the PCR mix. (Refs 80, 81) Dilution also reduces the concentration of competing RT primers with PCR primers. (Ref. 82) It is important to note that the RT and qPCR steps are separate and are not done in a single pot. The amplified products can be detected and quantified using SYBR green fluorescent molecules, which bind to any double-stranded DNA (dsDNA) [Figure 5Ai] or a fluorescent probe [Figure 5Aii].

Lin (Ref. 83) used stem-loop RT-qPCR with SYBR green fluorescence to detect three miRNAs in serum samples of stage IV lung cancer patients. (Ref. 83) Similarly, Resnick (Ref. 84) used a probe-based fluorescence detection method to detect six miRNAs in serum samples of ovarian cancer patients (Ref. 84) Zhang (Ref. 41) utilised stem-loop RT-qPCR to detect upregulated urinary extracellular vesicle miRNAs in early-stage renal cell carcinoma patients. (Ref. 41) (Ref. 46) reported detecting a polyomavirus-related miRNA in saliva and plasma from renal transplant patients using stem-loop RT-qPCR. (Ref. 46) Currently, there are two commercially available RT-qPCR kits for detecting microRNAs in liquid biopsies: Applied Biosystems’ TaqMan primer-probe assay and Qiagen’s miRCURY locked nucleic acid (LNA) assay [Table 1].

#### Poly(A)-tailing RT-qPCR

Instead of using stem-loop primers for RT, in poly(*A*)-tailing RT-qPCR, the 3′ end of the target miRNA sequence is polyadenylated. Subsequently, a universal RT primer transcribes the cDNA template before PCR amplification [Figure 5B] (Ref. 85). Almohsen (Ref. 86) used poly(A) tailing with SYBR green fluorescence to detect significant differences in the expression of target miRNAs from platelet-poor plasma in patients with acute myeloid leukaemia. (Ref. 86) Wan (Ref. 45) have also successfully used poly(A)-tailing RT-qPCR to detect HPV-positive head and neck squamous cell carcinomas from saliva samples. (Ref. 45) It is worth noting that sequence-specific and universal probes can be utilised for detecting amplified products in poly(A)-tailing RT-qPCR (Ref. 87).

#### Other thermal amplification methods

Xue (Ref. 82) reported a sensitive and multiplexed one-step RT-qPCR (SMOS-qPCR) technique for detecting target miRNAs, validated with serum samples of oesophagal cancer patients. (Ref. 85) SMOS-qPCR combines RT and PCR into a single step by utilising an extended primer for RT, which enables efficient binding and a higher reverse transcription rate [Figure 5C]. Compared to stem-loop RT-qPCR, which uses a lower primer concentration in the mix due to competition concerns during PCR, SMOS-qPCR uses a larger sequence that enhances reverse transcription efficiency. (Ref. 82) Among the novel detection methods discussed in this review, this system achieved the lowest reported LOD of 0.1 zM. (Ref. 85)

Droplet digital PCR (ddPCR) is an ultra-precise detection method used by Cirillo (Ref. 88) to detect signature miRNAs in serum samples of ovarian cancer patients. (Ref. 88) Unlike regular PCR, which amplifies nucleic acids in a single reaction, ddPCR partitions DNA samples into tens of thousands of water-in-oil nanodroplets, which all undergo PCR separately yet simultaneously in a single container [Figure 5D] (Ref. 89). Although this workflow requires a cumbersome setup, it effectively determines the absolute quantity of miRNA. (Refs 90, 91) Similarly, Torii (Ref. 92) successfully used ddPCR to detect miRNA from urine exosomes in infants with congenital cytomegalovirus. (Ref. 92) When comparing the performance of ddPCR and RT-qPCR for detecting target miRNA, RT-qPCR shows greater sensitivity than ddPCR, especially at low concentrations. This further emphasises the need to optimise ddPCR workflows before their widespread implementation in clinical settings. (Refs 93, 94)

### Isothermal amplification

Isothermal amplification methods are continuously being developed, which, unlike PCR, occur at a constant temperature. The primary advantages of isothermal amplification include eliminating costly thermal cycling equipment and its suitability for POC applications. (Ref. 95) However, in general, they are still limited by their relatively lower specificity levels, which can be attributed to their low amplification temperatures. (Ref. 95)

#### Rolling circle amplification

**Figure 6.**
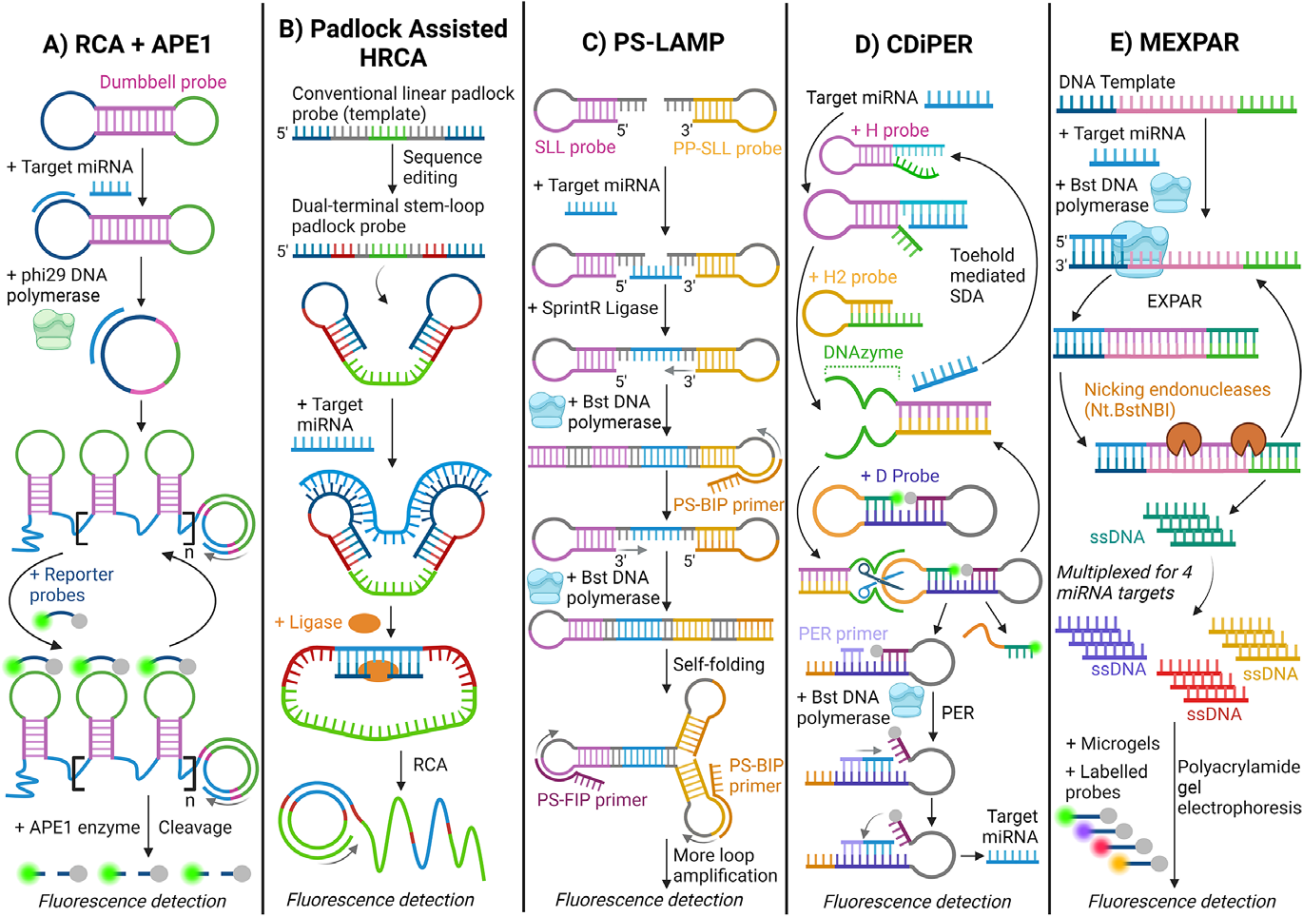
Schematic illustration of isothermal amplification methods for detecting miRNA. A. Rolling circle amplification (RCA) + APE1 enzyme; (Ref. 69) B. Padlock-assisted hyperbranched rolling circle amplification (HRCA); (Ref. 98) C. Primer-based loop-mediated isothermal amplification (PS-LAMP); (Ref. 100) D. Catalytic assembly of DNAzyme integrated with primer exchange reaction (CDiPER); (Ref. 101) and E. Multiplexed exponential amplification reaction (MEXPAR). (Ref. 42)

One of the most common isothermal amplification techniques is rolling circle amplification (RCA). (Ref. 96) Here, a circular DNA probe binds to the target miRNA, forming a template for continuous DNA synthesis and amplifying the miRNA sequence. (Ref. 97) Xie (Ref. 69) have reported the successful use of apyrimidinic endonuclease 1 (APE1) assisted RCA in dual-signal amplification to detect a target miRNA from serum samples in Alzheimer’s patients. (Ref. 69) The RCA + APE1 system used two probes: a dumbbell-shaped probe (DP) and a reporter probe (RP), where the sealed DP transforms into an active circular structure in the presence of target miRNA and phi29 DNA polymerase [Figure 6A]. Xu (Ref. 98) have also successfully detected miRNA in clinical samples using a reconstructed conventional linear padlock probe to improve the performance of RCA. (Ref. 98) By creating two stem-loops at the terminal ends of the padlock probe, the target recognition is enhanced by triggering a hyperbranched RCA (HRCA) reaction [Figure 6B].

#### Loop-mediated isothermal amplification

Loop-mediated isothermal amplification (LAMP) is another amplification technique in which four to six primers are used to recognise the target miRNA sequence. (Ref. 99) Talap (Ref. 100) have reported a modified ligation-initiated phosphorothioate primer-based LAMP (PS-LAMP) strategy for target miRNA detection from papillary thyroid cancer patients. (Ref. 100) This method involves ligating two linker probes, stem-loop linker (SLL) and phosphorylation stem-loop linker (PP-SLL), with the target miRNA to obtain an active amplicon. Strand-displacing Bst DNA polymerase initiates the amplification using two inner primers (PS-FIP/BIP) to form a loop structure. The remaining loop primers then initiate further cyclic amplification cycles. Both methods utilise probes with fluorescence and quencher to detect amplification rate [Figure 6C] (Ref. 99).

#### Other enzyme-based isothermal amplification methods

Other enzyme-based isothermal amplification methods were also explored for detecting miRNAs. For example, Zhang (Ref. 101) introduced a novel isothermal amplification method: catalytic DNAzyme integrated with primer exchange reaction (CDiPER) for miRNA detection in diabetes mellitus patients. (Ref. 101) In CDiPER, the target miRNA binds to hairpin probes (H/H2 probe), inducing conformational changes to form a DNAzyme. DNAzymes are single-stranded DNA molecules with catalytic function, and here, they cleave the double-loop probe (D probe), which activates the primer exchange reaction (PER), whereby primers are exchanged to amplify the fluorescence signals [Figure 6D].

Yao (Ref. 42) reported a cascade-based multiplexed exponential isothermal amplification reaction (MEXPAR) to simultaneously detect multiple urological target miRNAs to diagnose prostate, bladder, and renal cell cancers. (Ref. 42) The miRNAs undergo four parallel EXPARs, utilising Bst DNA polymerase and nicking endonucleases to release single-strand DNA (ssDNA), which is hybridised with microgels functionalised with fluorescence-labelled DNA probes [Figure 6E].

#### CRISPR/Cas-assisted isothermal amplification

Innovative miRNA detection systems integrating isothermal amplification and CRISPR technology have effectively distinguished various diseases from clinical samples. CRISPR, or clustered regularly interspaced short palindromic repeats, is a precise gene-editing tool that efficiently modifies targeted DNA sequences and has been successfully adapted for disease detection applications. (Ref. 102) These mechanisms integrate CRISPR-associated (Cas) enzymes, guided by synthetic RNA, to locate and cleave specific DNA sequences. (Ref. 103) This enables targeted genetic modifications through insertions, deletions, or alterations. (Ref. 103)

**Figure 7.**
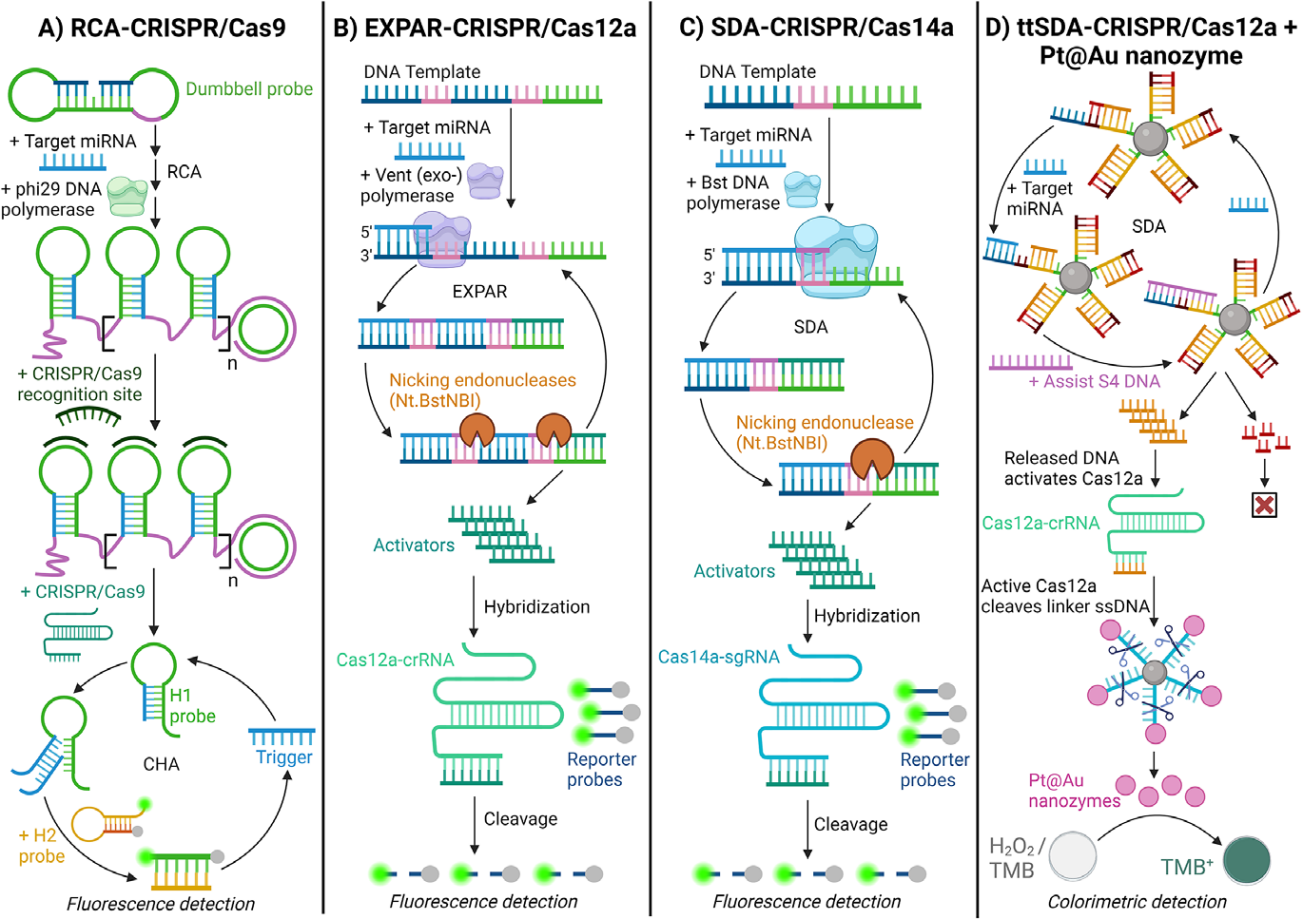
Schematic illustration of isothermal amplification methods with CRISPR/Cas systems for detecting miRNA. A. Rolling circle amplification (RCA)-CRISPR/Cas9; (Ref. 104) B. exponential amplification reaction (EXPAR)-CRISPR/Cas12a; (Ref. 105) C. Strand displacement amplification (SDA)-CRISPR/Cas14a; (Ref. 107) and D. Target-triggered SDA (ttSDA)-CRISPR/Cas12a + Pt@Au nanozymes. (Ref. 108)

Liu (Ref. 104) presented a combination of RCA and CRISPR/Cas9 for miRNA detection. (Ref. 104) In this method, the target miRNA hybridises with the ends of a dumbbell probe, forming a cyclised padlock structure. This padlock then undergoes RCA, facilitated by phi29 DNA polymerase, to produce ssDNA with repeated hairpin structures. The ssDNA is hybridised with the CRISPR/Cas9 recognition site, which the CRISPR/Cas9 system cleaves, disassembling the RCA product into hairpin probes (H1 probes). The target miRNA then unfolds the H1 probes, initiating a catalytic hairpin assembly (CHA) process that generates a detectable fluorescence signal through binding with a fluorescence-labelled H2 probe [Figure 7A] (Ref. 104).

Similarly, Yang (Ref. 105) used EXPAR and CRISPR/Cas12a to detect miRNAs. Yang (Ref. 105) reported that CRISPR/Cas12a has a more sensitive detection profile when coupled with EXPAR, as opposed to using CRISPR/Cas12a as a standalone assay. (Ref. 105) The target miRNA has a complementary template strand containing binding regions for an endonuclease-nicking enzyme and a CRISPR/Cas12a activator. With vent(exo-) polymerase, the EXPAR releases activators that bind to Cas12a-CRISPR RNA (crRNA). The volume of the activator that is repeatedly produced from the amplification reaction prompts Cas12a to trans-cleave a quenched fluorescence reporter probe, leading to an amplified fluorescent signal [Figure 7B] (Ref. 105).

Strand displacement amplification (SDA) is an isothermal technique that uses four primers to initiate a cyclic reaction, in addition to restriction enzymes and endonucleases, to target recognition and cleavage sites. (Ref. 106) DNA polymerase binds at the nicked site, allowing strand extension and displacement, leading to exponential amplification. (Ref. 106) Chi (Ref. 107) used this technique to detect circulating miRNA from serum by integrating it with CRISPR/Cas14a. (Ref. 107) Here, a template DNA binds with the target miRNA at its recognition site, and an endonuclease nicks the Cas14a activation site of the template DNA, releasing activators that bind to the CRISPR/Cas14a-single guide RNA (sgRNA) and allow Cas14a to trans-cleave quenched fluorescence reporter probes, amplifying the fluorescent signal [Figure 7C] (Ref. 107).

Luo (Ref. 108) used a similar method of SDA and a nanozyme-mediated CRISPR-Cas12a system for miRNA detection in hepatocellular carcinoma patients. (Ref. 108) This method employed toehold-triggered SDA, which bypasses the need for enzymes. Here, the target miRNA and assisting single-stranded DNA_4_ (S4) hybridise with exposed toehold regions on custom magnetic beads, triggering the SDA reaction and releasing DNA complementary to the crRNA, activating the CRISPR-Cas12a system. The active Cas12a cleaves ssDNA linkers, releasing Pt@Au nanozymes from magnetic beads. The released nanozymes, which possess peroxidase-like activity, catalyse 3,3′,5,5′-tetramethylbenzidine (TMB) oxidation by hydrogen peroxide (H_2_O_2_). This reaction causes a colour change that can be visually observed or measured using an ultraviolet–visible spectrophotometer, where the intensity of the colour change positively correlates with the concentration of the target miRNA [Figure 7D] (Ref. 108).

Furthermore, alternative CRISPR-based systems for miRNA detection, particularly those utilising Cas13, have been demonstrated. Notably, the CRISPR/Cas13a-based workflows were capable of ultra-sensitive miRNA detection (4.5aM—2.6fM), exhibiting impressively low detection limits when tested with miRNA spiked in human liquid biopsy samples. (Refs 109, 110, 111) CRISPR/Cas amplification systems are also being explored, along with innovative light-activation mechanisms to enhance signal amplification. A recent study demonstrated a light-activated CRISPR/Cas12a amplification system that successfully detected miRNA21 in MCF-7 cancer cell lines (Ref. 112).

#### Isothermal amplification methods using nanomaterials

**Figure 8.**
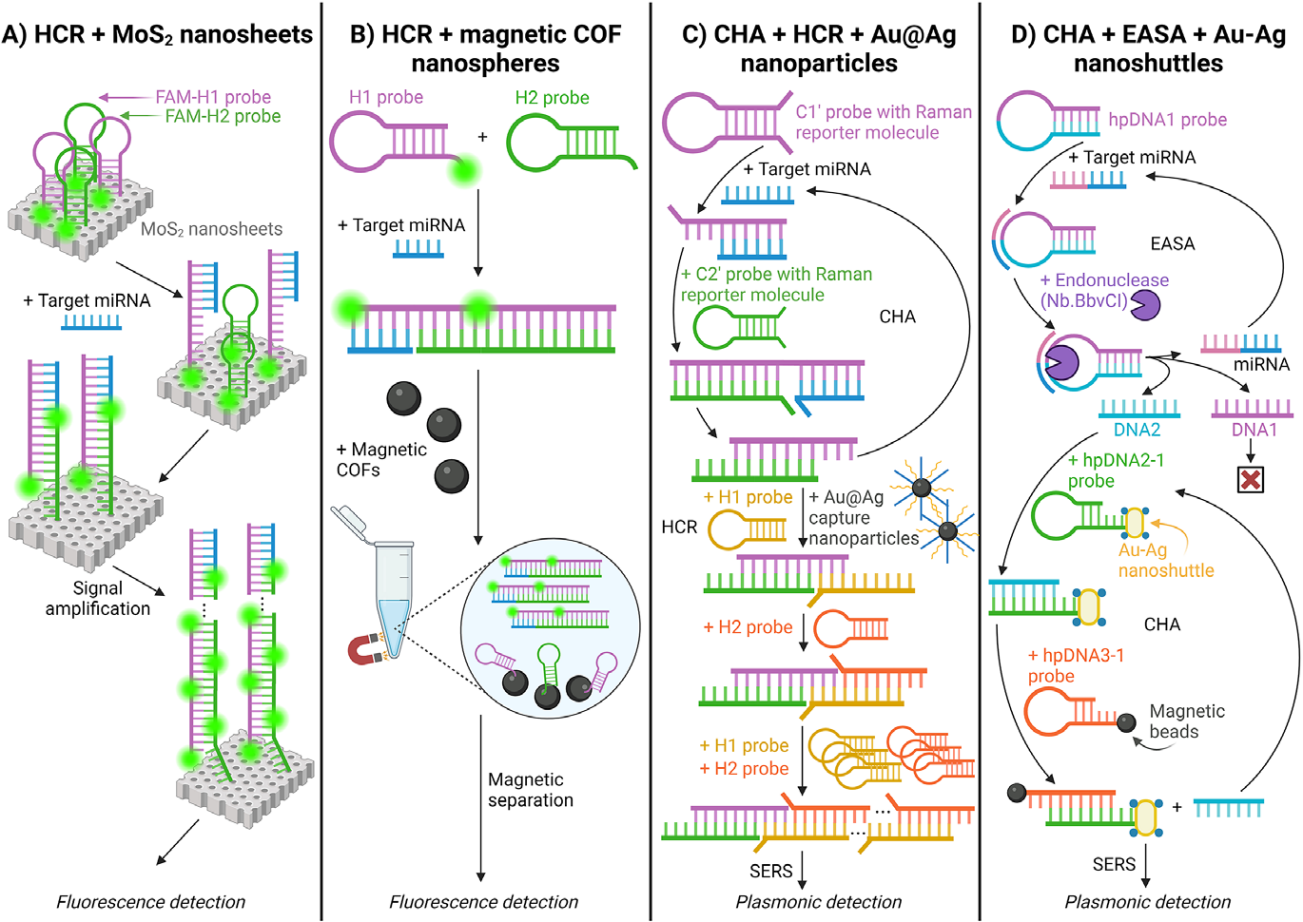
Schematic illustration of isothermal amplification methods with nanomaterials for detecting miRNA. A. Hybridisation chain reaction (HCR) + Molybdenum disulfide (MoS_2_) nanosheets; (Ref. 113) B. HCR + magnetic covalent organic framework (COF) nanospheres; (Ref. 116) C. Catalytic hairpin assembly reaction (CHA) + HCR + Au-Ag nanoparticles; (Ref. 117) and D. CHA + enzyme-assisted signal amplification (EASA) + Au–Ag nanoshuttles. (Ref. 72)

Various miRNA detection systems that incorporate isothermal amplification methods with nanomaterials to modify or amplify the reaction have been developed. One example can be found in the research of Wei (Ref. 113), whose research used hybridisation chain reaction (HCR) with molybdenum disulfide (MoS_2_) nanosheets in plasma-derived exosomes from lung cancer patients. (Ref. 113) HCR uses specifically designed DNA hairpins to amplify target-specific signals without enzymes. (Refs 114,115) The process starts with the adsorption of fluorescently-labelled DNA probes (FAM-H1 and FAM-H2) onto the MoS_2_ nanosheets, resulting in fluorescence quenching. The presence of target miRNA initiates the HCR by binding to FAM-H1, triggering a cascade of hybridisation events between FAM-H1 and FAM-H2. This forms long dsDNA products that detach from the nanosheets, leading to amplified signals and fluorescence detection [Figure 8A] (Ref. 113).

Similarly, Liang (Ref. 116) used HCR with magnetic covalent organic framework (COF) nanospheres to detect miRNA in glioma patients. (Ref. 116) The nanospheres consist of Fe_3_O_4_ nanoparticles and high-crystalline COF shells. When target miRNA is absent, the two hairpin DNA probes, H1 and H2, are adsorbed onto the nanospheres, resulting in quenched fluorescence. However, when miRNA is present, the hairpin probes hybridise with the miRNA, creating non-adsorbable dsDNA, thus blocking the fluorescence quenching [Figure 8B]. Therefore, the change in fluorescence intensity reflects the target miRNA concentration. (Ref. 116)

Chen (Ref. 117) developed a surface-enhanced Raman spectroscopy (SERS) biosensor to detect miR-210 in blood erythrocytes using two signal amplification strategies: CHA and HCR. The system employs four Raman reporter-labelled probes. (Ref. 117) When the target miRNA binds to the C1’ probe, the C2’ probe binds and subsequently releases the miRNA. With the addition of Au@Ag nanoparticles and H1 & H2 probes, HCR is triggered, causing consecutive and continuous binding of H1/H2 probes onto the sticky ends of the C1’/C2’ double-strand. The Au@Ag nanoparticles cause aggregation of Raman reporters, generating a detectable SERS signal [Figure 8C] (Ref. 117).

Dai (Ref. 72) introduced a highly sensitive version of CHA combined with enzyme-assisted signal amplification (EASA). (Ref. 72) In EASA, the target miRNA binds to the looped section of the hairpin DNA1 (hpDNA1) probe, which contains an endonuclease recognition site. The endonuclease cleaves the hairpins into DNA1 and DNA2, leaving the target miRNA intact for repeated EASA cycles. DNA2 binds to a hairpin DNA2–1 (hpDNA2–1) probe labelled with Au–Ag nanoshuttles, serving as the SERS nanoprobes. This structure further hybridises with a hairpin DNA3 (hpDNA3) probe labelled with magnetic beads, which act as capture nanoprobes. The magnets allow aggregation of the complexes, further amplifying the SERS signal [Figure 8D] (Ref. 72).

#### Isothermal amplification methods using metal–organic frameworks

Metal–organic frameworks (MOFs) are increasingly integrated into miRNA detection systems to enhance isothermal amplification strategies. This novel class of self-assembling crystalline materials offers unique advantages. (Ref. 118) Their popularity in miRNA detection stems from their adjustable porosity, expansive surface area, customisable chemistry, thermal resilience, and luminescent properties. (Ref. 119)

**Figure 9.**
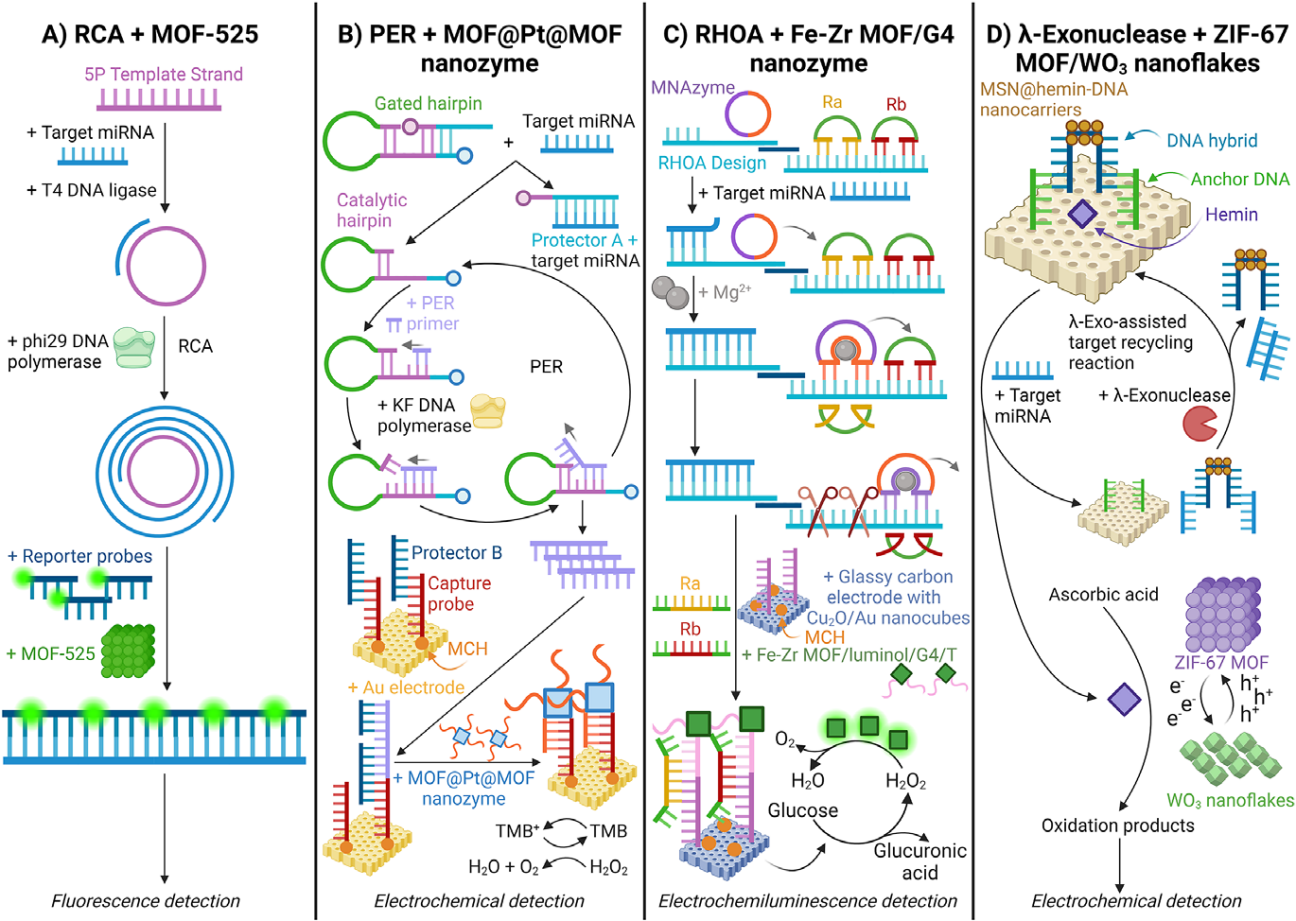
Schematic illustration of isothermal amplification methods with metal–organic frameworks (MOFs) for detecting miRNA. A. Rolling circle amplification (RCA) + MOF-525; (Ref. 120) B. Primer exchange reaction (PER) + MOF@Pt@MOF nanozyme; (Ref. 121) C. Rolling hoop orbital amplification (RHOA) + Iron-Zirconium (Fe-Zr) MOF/G-quadruplex (G4) nanozyme; (Ref. 122) and D. λ-Exonuclease + ZIF-67 MOF/ Tungsten(VI) oxide (WO_3_) nanoflakes. (Ref. 123)

Sun (Ref. 120) developed a miRNA detection method using MOF-525, a self-fluorescent framework, and target-triggered RCA for colorectal cancer patients from serum exosomes. (Ref. 120) Here, the target miRNA hybridises with a template strand, initiating RCA via phi29 DNA polymerase. The amplified product then forms dsDNA with fluorescent reporter probes, preventing their adsorption and quenching by MOF-525. This results in a change in the fluorescence ratio that correlates positively with the target miRNA concentration, enabling sensitive and specific detection [Figure 9A] (Ref. 120).

Li (Ref. 121) successfully integrated isothermal amplification with MOFs and nanomaterials, developing a PER-combined MOF@Pt@MOF nanozyme. (Ref. 121) Nanozymes are nanoparticles with enzyme-like catalytic properties. (Ref. 121) The MOF nanozyme consists of Pt nanoparticles sandwiched between MIL-88 and coated with ssDNA. It utilises a gated hairpin that activates upon hybridisation with target miRNA, splitting into catalytic and protector A probe-miRNA complexes. The catalytic hairpin initiates PER with primers and KF DNA polymerase, releasing ssDNA. This ssDNA binds to a protector B probe on an Au electrode functionalised with 6-mercapto-1-hexanol (MCH), displacing multiple protector B probes. The nanozyme’s ssDNA then hybridises with the capture probe on the electrode, catalysing TMB oxidation by H_2_O_2_ and generating an amplified electrochemical signal [Figure 9B] (Ref. 121).

Another notable isothermal amplification technique is rolling hoop orbital amplification (RHOA) - a technique used by Gao (Ref. 122). Here, RHOA was combined with an iron-zirconium (Fe-Zr) MOF/G-quadruplex (G4) nanozyme with peroxidase activity. When the target miRNA binds to the RHOA design, the multiplexed nucleic acid enzyme (MNAzyme) is displaced by sequentially opening circular DNA probes (Ra and Rb). The process is initiated as the MNAzyme amplifies and cleaves the Ra probe in the presence of magnesium ions (Mg^2+^), which is repeated with the Rb probe. As a result, many Ra and Rb probes bind to the Fe-Zr MOF/G4 nanozyme, which is hybridised onto a glassy carbon electrode with copper (I) oxide (Cu_2_O)/Au nanocubes. After adding glucose to the system, oxidation by Cu_2_O/Au generates H_2_O_2_ and glucuronic acid. The MOF catalyses the decomposition of H_2_O_2_, which in turn reacts with luminol, producing a detectable signal via electrochemiluminescence [Figure 9C] (Ref. 122).

Platform-based isothermal amplification assisted by λ-exonuclease (λ-exo) has also been researched by Wang (Ref. 123) for the detection of exosomal miRNA. (Ref. 123) Here, mesoporous silica nanoparticles (MSNs) trap hemin nanocarriers, which quench photocurrent upon introducing the target miRNA. The release of hemin is prompted by the displacement of miRNA from the DNA hybrid on the MSN surface. A novel ZIF-67 MOF decorated with tungsten (VI) oxide (WO_3_) nanoflakes is utilised to enhance the photoresponse during the reaction by facilitating charge separation correlated with the abundance of target miRNA. With hemin, the electrons from the MOF are transferred to the WO_3_ nanoflakes, using ascorbic acid as the electron donor [Figure 9D] (Ref. 123). The oxidised products can then be detected electrochemically.

### Hybridisation

A significant advance in miRNA detection, specifically towards POC testing, has been the development of detection platforms that bypass signal amplification. Several different amplification-free hybridisation methods have been successfully validated with clinical patient samples.

#### Nanomaterial biosensors

**Figure 10.**
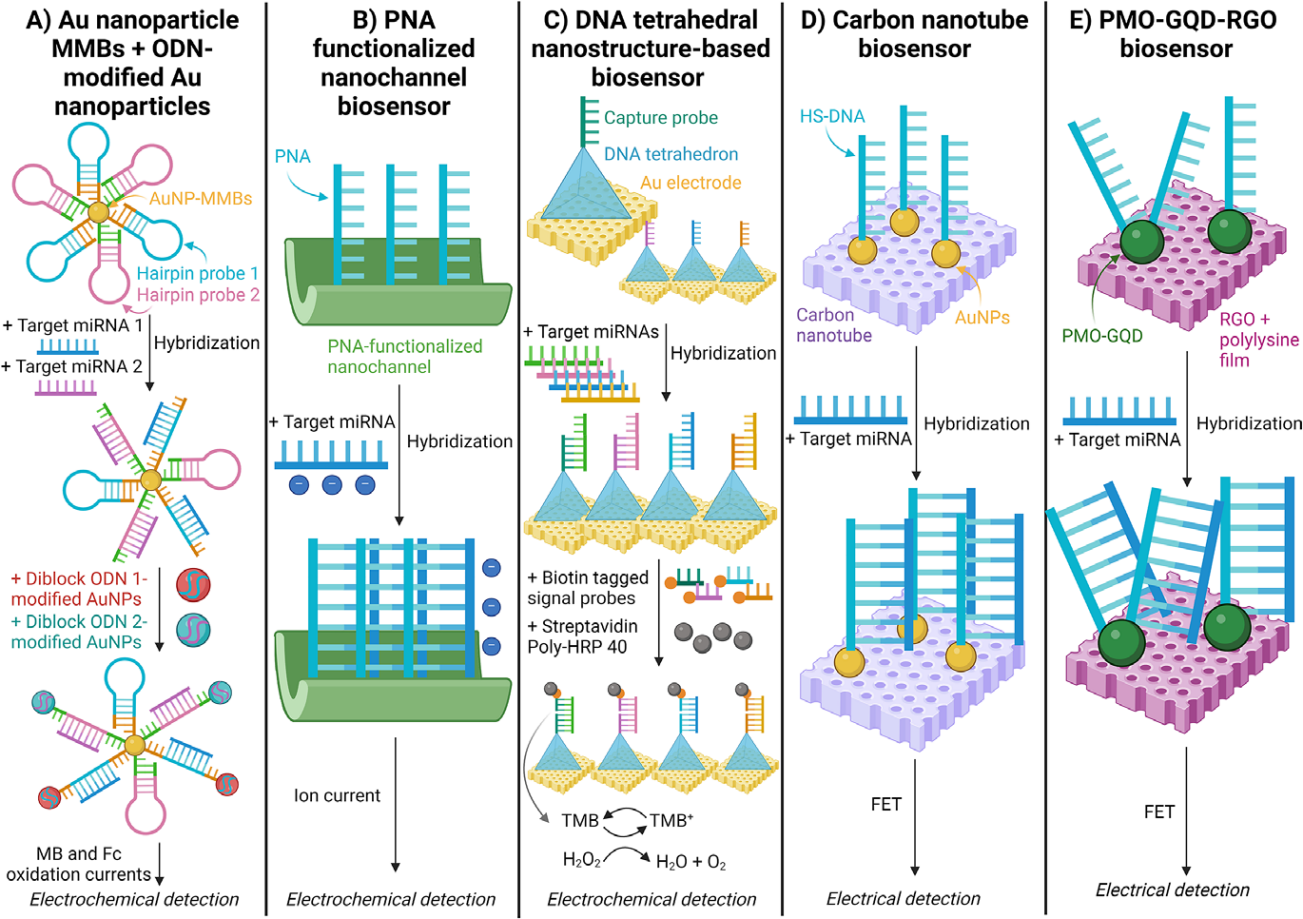
Schematic illustration of electrochemical and electrical nanoparticle miRNA detection methods. A. Magnetic microbeads & diblock oligonucleotide-modified Au nanoparticles; (Ref. 70) B. Peptide nucleic acid (PNA) functionalised nanochannel biosensor; (Ref. 124) C. DNA tetrahedral nanostructure-based biosensor; (Ref. 125) D. Carbon nanotube biosensor; (Ref. 126) and E. Phosphorodiamidate morpholino oligomers (PMO) - graphene quantum dots (GQDs) - functionalised reduced graphene oxide (RGO) field effect transistor (FET) biosensor. (Ref. 127)

Previously, nanomaterials were described in conjunction with isothermal amplification. However, these materials have also been applied successfully in amplification-free methods. For example, Wang (Ref. 70) used gold-coated magnetic microbead nanoparticles (AuNP-MMBs) with diblock oligonucleotide (ODN)-modified AuNPs to detect serum-derived miRNAs in glioma patients. (Ref. 70) Two target miRNAs were hybridised on two respective hairpin probes on the surface of AuNP-MMBs, allowing attachment of the diblock-ODN modified AuNPs to the end of the hairpin probe [Figure 10A]. The abundance of target miRNA was quantified by measuring the oxidation peak currents of methylene blue (MB) and Ferrocene (Fc) moieties present on the diblock-ODN modified AuNPs. (Ref. 70)

Xiao (Ref. 124) used a unique biosensing technique to detect miRNAs associated with pancreatic cancer. (Ref. 124) They employed a peptide nucleic acid (PNA) functionalised nanochannel to detect exosomal miR-10b. The PNA was covalently bound to nanochannels, where extracted exosomal miRNAs were hybridised to the surface PNAs. Due to the negatively charged phosphate backbone of miRNA, the ionic current was measured and correlated to the abundance of miRNA in the sample [Figure 10B] (Ref. 124).

Zeng (Ref. 125) reported multiplexed simultaneous detection of target miRNAs associated with overexpression in pancreatic carcinoma patients using a nanostructure-based electrochemical biosensor. (Ref. 125) DNA tetrahedral nanostructures are bound with a capture probe on an Au electrode. Subsequently, the target miRNA hybridises with the capture probe on the nanostructure, and a biotin-tagged signal probe facilitates the binding of streptavidin poly-HRP40, a biotin-binding protein. This catalyses the reduction of H_2_O_2_ in the presence of the TMB substrate, generating a measurable amperometric signal [Figure 10C] (Ref. 125).

Li (Ref. 126) have demonstrated the use of field-effect transistor (FET) biosensors based on a carbon nanotube (CNT) film for detecting exosomal miRNA in breast cancer patients with a highly sensitive detection limit of 0.87 aM. (Ref. 126) Here, thiolated DNA probes (HS-DNA) are immobilised on the surface of AuNPs. When the DNA probe hybridises with the target miRNA, it introduces additional negative charges at the sensing interface, leading to p-type electrostatic doping, measured by the FET sensor [Figure 110D].

Li (Ref. 127) also demonstrated a similar case of exosomal miRNA detection in breast cancer patients using surface probe hybridisation and electrical detection. (Ref. 127) This technique used a phosphorodiamidate morpholino oligomers-graphene quantum dots (PMO-GQD) complex covalently bound to a reduced graphene oxide (RGO) FET [Figure 10E]. Here, the miRNA changes the net carrier density, which is similarly measured by the FET sensor.

**Figure 11.**
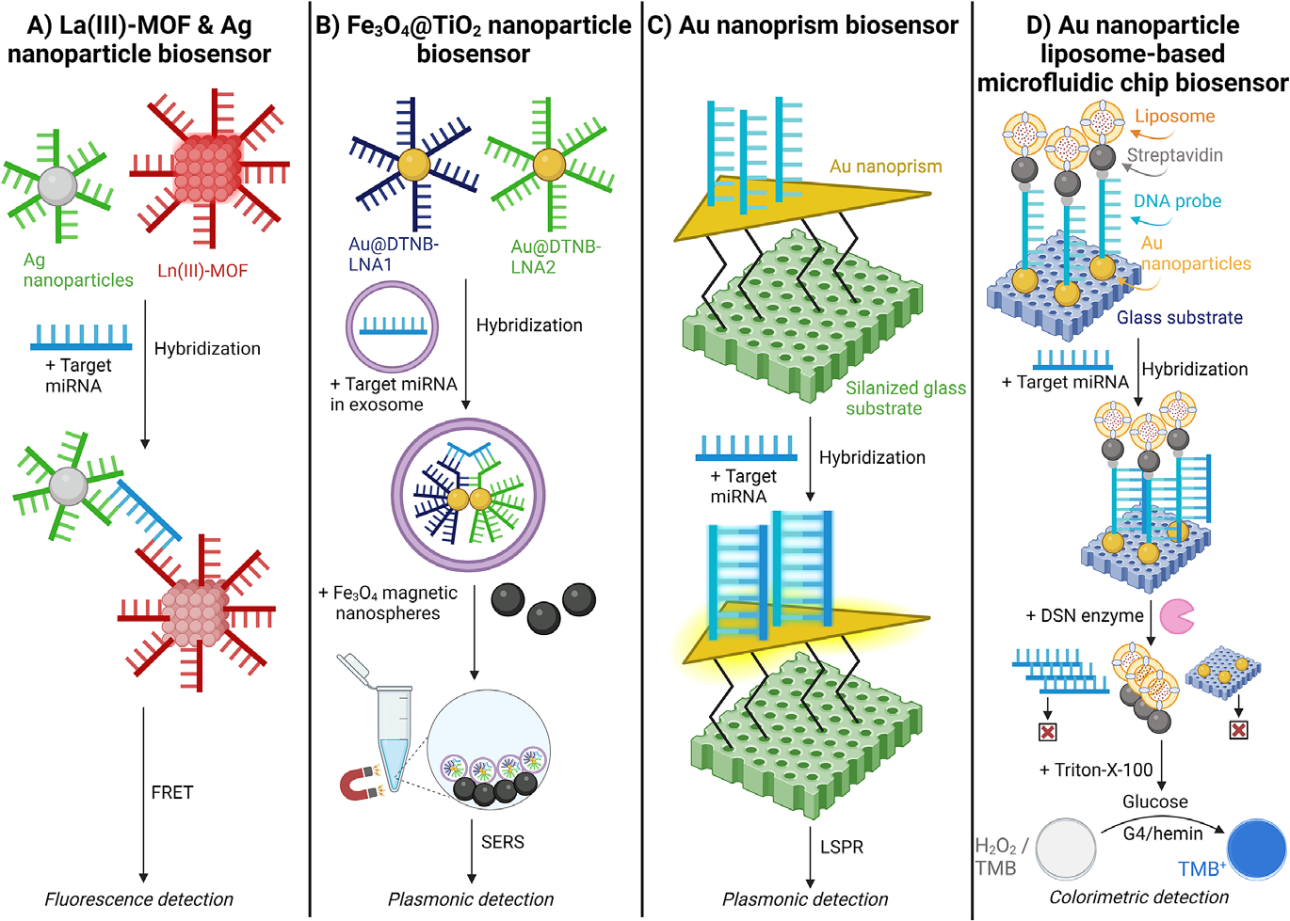
Schematic illustration of fluorescent, plasmonic, and colourimetric nanoparticle miRNA detection methods. A. Lanthanum oxide (La(III))-metal–organic framework (MOF) & silver (Ag) nanoparticle biosensor; (Ref. 128) B. Iron(II,III) oxide@Titanium oxide (Fe_3_O_4_@TiO_2)_ nanoparticle biosensor; (Ref. 129) C. Gold (Au) nanoprism biosensor;(Ref. 71) and D. Gold (Au) nanoparticle liposome-based microfluidic chip biosensor. (Ref. 130)

Afzalinia and Mirzaee (Ref. 128) used a sandwich-type hybridisation biosensor to detect lung and breast cancer from plasma samples. (Ref. 128) This study used La(III)-MOF and Ag nanoparticles to hybridise with the target miRNA. Upon hybridisation, the La(III)-MOF and Ag nanoparticles function as energy donor-acceptor pairs in the fluorescence resonance energy transfer (FRET) process. The presence of the target miRNA induces FRET, leading to the quenching of the fluorescent signal [Figure 11A] (Ref. 128).

Jiang (Ref. 129) utilised a locked nucleic acid (LNA)-modified Au@DTNB (Raman reporter molecule) SERS tags for epithelial ovarian cancer detection. (Ref. 129) The LNA provides increased stability during high temperatures and consistent affinity for the target miRNA. The Au@DTNB SERS tags enter the exosomes and hybridise with the target miRNA. Subsequently, magnetic Fe_3_O_4_@TiO_2_ nanoparticles capture the SERS tags, enriching the exosomes and further amplifying the SERS signal [Figure 11B] (Ref. 129).

Joshi (Ref. 71) demonstrated a label-free detection of miRNA by utilising an ultrasensitive localised surface plasmon resonance (LSPR)-based sensor validated with pancreatic ductal adenocarcinoma and chronic pancreatitis patient plasma samples. (Ref. 71) Target miRNA complementary ssDNA probes were immobilised on Au nanoprisms. Here, the hybridisation of target miRNA with the ssDNA probes increases the local refractive index around the nanoprisms, resulting in a shift of the LSPR peak wavelength. The LSPR-based sensor was highly specific, distinguishing miR-10b from its single nucleotide variant, miR-10a [Figure 11C] (Ref. 71).

Zeng (Ref. 130) tested a liposome-based microfluidic platform to detect miRNA differences in type II diabetic patients. (Ref. 130) This biosensor immobilised Au nanoparticles on a glass surface, which hybridised with a DNA probe modified with thiol groups and biotin. The DNA probe then immobilises biotin-modified liposomes encapsulating glucose oxidase. Following hybridisation with the target miRNA, a duplex-specific nuclease (DSN) re-releases the liposomes. The contents of the liposome are released, causing a colour change due to H_2_O_2_ decomposition and TMB oxidation, catalysed by G4/hemin [Figure 11D] (Ref. 130).

#### Microarray

**Figure 12.**
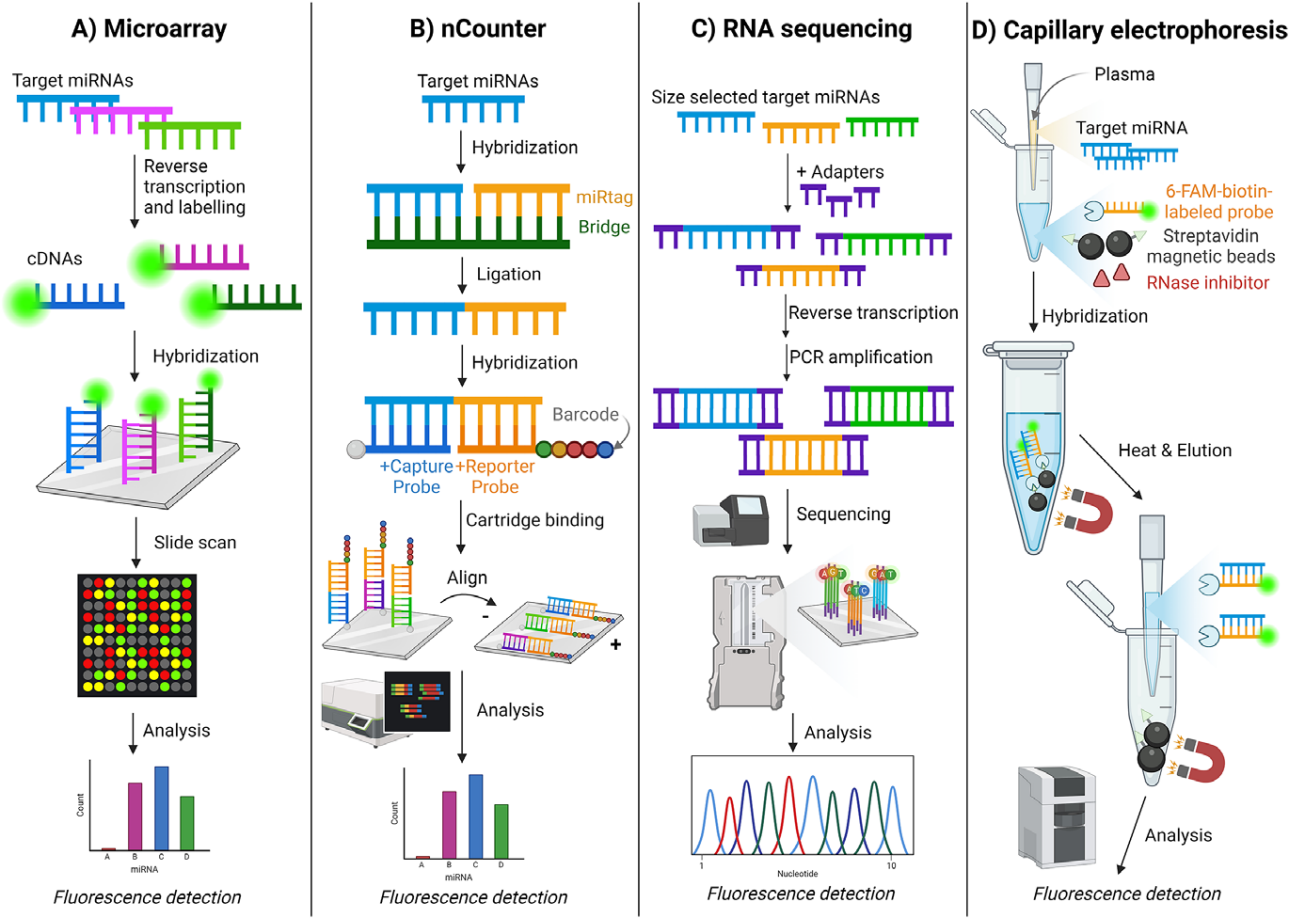
Schematic illustration of various miRNA detection methods. A. Microarray, (Ref. 132) B. nCounter, (Refs 43, 134) C. RNA sequencing, (Ref. 137) and D. Capillary electrophoresis. (Ref. 145)

A microarray is a unique multiplexing technique for miRNA detection. However, it has a considerably lower throughput than the RNA sequencing detection method but a higher throughput than the typical qPCR method. (Ref. 131) In microarrays, the target miRNA is transcribed to cDNA and labelled with fluorescent markers. These signature cDNA strands hybridise onto probes on the microarray surface, creating a cluster of hybridised samples that fluoresce when scanned, leading to a distinct and detectable array [Figure 12A]. Researchers Zhang and Hu (Ref. 132) used a microarray slide that can target an extensive list of miRNAs: a 3D-gene human miRNA oligo chip. (Ref. 132) This microarray was used for early detection of various cancers, targeting 50 different signature miRNAs. The study reported detection sensitivity >99% in >1,000 serum samples from patients with stage 1 lung, biliary tract, bladder, colorectal, oesophagal, gastric, glioma, liver, pancreatic, sarcoma, breast and prostate cancers. (Ref. 132)

#### nCounter

The nCounter (Nanostring) system can directly detect miRNA from the isolation step in a multiplex fashion. Like microarrays, it immobilises target miRNAs on a treated surface to identify multiple targets simultaneously with unique codes. However, unique to nCounter is the ligation of the target miRNA to miRtags and a bridge oligonucleotide sequence. The resulting complex hybridises with a ligand (biotin) for immobilisation and a target-specific barcode that generates a unique sequence of fluorophores. The complex is immobilised on the surface and quantified for each signature miRNA complex using Nanostring’s Digital Analyser [Figure 12B] (Ref. 133). Niemira (Ref. 134) successfully used nCounter to detect two miRNAs in serum from high-grade serous ovarian cancer patients. (Ref. 134) Additionally, Shankar (Ref. 43) reported successful miRNA detection in urinary exosomal miRNA expression levels in patients with IgA nephropathy with 100% sensitivity and specificity. (Ref. 43)

### RNA Sequencing

Next-generation sequencing (NGS) is widely used for large-scale nucleotide sequencing and diagnostics. RNA sequencing identifies and quantifies RNA molecules in a sample, offering a comprehensive view of the transcriptome and enabling the detection and measurement of various RNA forms. (Ref. 135) NGS has benefits such as low cost at pooled samples, multiplexed testing, and higher throughput. Still, it has notable drawbacks, including extensive sample preparation requirements compared to other nucleic acid detection methods and unsuitability for POC testing. (Ref. 5,136)

Geng (Ref. 137) used the MiSeqDx (Illumina) NGS instrument to detect multiple miRNAs simultaneously in adenocarcinoma and squamous cell carcinoma patients from plasma samples. (Ref. 137) In this method, small RNA molecules, including miRNAs, are isolated from a sample. These RNAs are ligated with 3′ and 5′ adapters and converted into cDNA. The cDNA is then amplified via PCR and purified, after which the cDNA libraries are sequenced. This allows for the identification and quantification of miRNAs [Figure 12C] (Ref. 137).

### Capillary Electrophoresis

Capillary electrophoresis (CE) is a common technique that separates ions by their movement under an applied voltage in tiny capillaries of micro- and nano-fluidic channels. (Ref. 138) CE is often utilised to separate and analyse proteins, peptides, and nucleic acids. (Refs 139–144) Ban (Ref. 145) developed a novel method combining magnetic bead-based CE with laser-induced fluorescence (CE-LIF) to detect miRNAs in lung cancer patients’ plasma samples successfully. (Ref. 145) This method used streptavidin-coated magnetic beads that hybridise with biotinylated fluorescent DNA probes (6-FAM-biotin-labelled probes) complementary to the target miRNA. An RNase inhibitor was added to reduce miRNA degradation by RNase in the plasma sample. After hybridisation, the miRNA-probe complexes are eluted by heating. The eluted complexes are then directly injected into the CE-LIF system for analysis [Figure 12D].

## Conclusion and Future Perspectives

The field of miRNA detection using liquid biopsies is rapidly evolving. Employing liquid biopsies minimises sample collection’s invasiveness and accelerates progress toward POC miRNA testing systems. These advancements are highly significant given the crucial role miRNAs play in gene regulation and disease progression. This analysis reviewed numerous miRNA workflows, detailing each step from sample collection and RNA isolation to amplification, processing, and detection methods. This review found that miRNAs can be effectively detected in clinical samples from blood, urine, and saliva - with blood being the most commonly used sample type. While some RNA isolation methods perform exceptionally well, they are unsuitable for high-throughput or POC testing, which are important considerations for advancing miRNA testing systems. Importantly, three studies successfully bypassed RNA isolation, achieving detection limits of 4.26 aM to 0.21fM, demonstrating the potential for future development and use. (Refs 70, 71, 72) In miRNA sample processing, many innovative techniques have been developed and successfully implemented. One example is SMOS-qPCR, which achieved the lowest reported LOD of 0.1 zM. (Ref. 85) However, as PCR methods are unsuitable for POC testing, ongoing advancements in alternative miRNA processing techniques are crucial. Thus, the wide range of isothermal amplification methods were detailed, either as standalone approaches or in combination with CRISPR/Cas systems, nanoparticles, or MOFs. Notably, processing methods that bypass amplification entirely - despite the challenge posed by the low abundance of miRNA in samples - further advance POC testing systems. Several amplification-free, hybridisation-based techniques demonstrated exceptionally low detection limits, with the lowest being 0.87 aM, as reported by Li (Ref. 126), using a carbon-nanotube FET. (Ref. 126) The emerging development of POC-compatible testing systems also holds significant potential for broader applications, including acute infectious disease monitoring and chronic disease management. (Ref. 146)

In addition to miRNA, several other blood-based biomarkers are being explored for cancer detection. For instance, protein biomarkers have well-established assays but often lack the sensitivity and specificity needed for precise detection. (Ref. 147) Additionally, circulating tumour DNA (ctDNA) enables mutation-specific monitoring and resistance profiling, though it requires timely sampling, is labour-intensive, and is costly. (Refs 147,148) Lastly, circulating tumour cells (CTC) show promise in early diagnosis and monitoring response to therapy, yet are also expensive and labour-intensive, and results can be impacted by CTC undergoing epithelial-mesenchymal transition. (Refs 147,148) Compared to other methods, in the landscape of liquid biopsy for cancer detection, miRNA has shown considerable promise. Its stability in blood, role as a gene regulator, and established detection and processing methods have contributed to its advancement in clinical applications. miRNA’s unique characteristics position it as a potentially valuable tool for early diagnosis and monitoring, though further research and validation are still needed to understand its clinical potential fully.

Given the role of miRNA dysregulation in cancer development, miRNAs are also being investigated for therapeutic applications. For example, instances of anti-microRNA formulations designed to block pathways associated with dysregulated miRNAs are also being explored by Yan et al (2023). (Ref. 149) Although current methods are limited by challenges like off-target effects and low cellular uptake, innovative strategies are still being explored to overcome these limitations. (Ref. 150)

This review discusses and highlights novel workflows for miRNA detection from liquid biopsies. The reviewed papers have validated their designs using clinical samples. However, many clinical studies were limited to small, controlled sample sizes, necessitating future validation in diverse, large patient cohorts.

A significant advancement in the field worth mentioning is the initiation of multiple clinical trials to detect miR-371a-3p in peripheral blood to diagnose stage 1 testicular germ cell cancer. For instance, one trial has progressed to enrolling patients for an observational cohort study across a spectrum of risk levels at various North American hospitals. (Refs 151–153) The detection of miR-371 in patient samples was successful both with RT-qPCR and ddPCR, although RT-qPCR detected the target miRNA with higher sensitivity than ddPCR. (Ref. 94)

The integration of miRNA-based diagnostic workflows into clinical practices demands the establishment of standardised protocols with normalisation factors. Often, these protocols should employ stable expression of reference miRNA across sample types and abide by strict benchmarks for target miRNA quantification. Hence, robust normalisation processes for endogenous genes or spike-in controls are necessary for comparing results and providing an accurate diagnosis of the pathophysiological state of the patient from miRNA for precise clinical intervention. The interpretation of the results, therefore, should also incorporate a grey zone, representing values that fall outside expected levels, to account for minor abnormalities in miRNA levels that do not require immediate clinical intervention intervention. (Refs 14,154)

Another challenge in developing miRNA detection systems is the significant cost variation of custom or off-the-shelf consumables, reagents, and instrumentation, highlighting the need for more cost-effective solutions to enhance the accessibility and adaptability of these technologies. (Ref. 155) Ongoing advancements to address challenges like high costs, limited sensitivity, and insufficient detection limits in novel miRNA detection methods are expected to pave the way for more reliable and accessible POC platforms. These developments are anticipated to enhance early disease diagnostics significantly.
